# Accelerating progress on early childhood development for children under 5 years with disabilities by 2030

**DOI:** 10.1016/S2214-109X(21)00488-5

**Published:** 2022-01-14

**Authors:** Global Research on Developmental Disabilities Collaborators, Bolajoko O Olusanya, Nem Yun Boo, M K C Nair, Maureen E Samms-Vaughan, Mijna Hadders-Algra, Scott M Wright, Cecilia Breinbauer, Nihad A Almasri, Marisol Moreno-Angarita, Jalal Arabloo, Narendra K Arora, Sandra S Block, Brad D Berman, Gwen Burchell, Olaf K de Camargo, Gwen Carr, Christie del Castillo-Hegyi, Vivian G Cheung, Ricardo Halpern, Rosa A Hoekstra, Paul Lynch, Mphelekedzeni C Mulaudzi, Angelina Kakooza-Mwesige, Felix A Ogbo, Jacob O Olusanya, Valeria Rojas-Osorio, Amira Shaheen, Andrew N Williams, Chiara Servili, Melissa Gladstone, Hannah Kuper, Donald Wertlieb, Adrian C Davis, Charles R J Newton

**Affiliations:** Centre for Healthy Start Initiative, Ikoyi, Lagos, Nigeria; Department of Population Medicine, Faculty of Medicine and Health Sciences, Universiti Tunku Abdul Rahman, Selangor, Malaysia; NIMS-Spectrum-Child Development Centre, NIMS Medicity Campus, Aralummoodu, Thiruvananthapuram, Kerala, India; Department of Child and Adolescent Health, The University of the West Indies, Mona Campus, Kingston, Jamaica; University of Groningen, University Medical Center Groningen, Department of Paediatrics, Division of Developmental Neurology, Groningen, Netherlands; Division of General Internal Medicine, Johns Hopkins Bayview Medical Center, Johns Hopkins University School of Medicine, Baltimore, MD, USA; Center for Healthy Development, Seattle, WA, USA; Department of Physiotherapy, The University of Jordan, Amman, Jordan; Department of Human Communication, Faculty of Medicine, National University of Colombia, Bogota, Colombia; Health Management and Economics Research Center, Iran University of Medical Sciences, Tehran, Iran; The INCLEN Trust International, Okhla Industrial Area (Phase 1), New Delhi, India; National Center for Children’s Vision and Eye Health, Chicago, IL, USA; UCSF Benioff Children’s Hospitals, Progressions: Developmental and Behavioral Pediatrics, Walnut Creek, CA, USA; United Aid for Azerbaijan, Hüseyn Cavid Prospekti, Azerbaijan; Centre for Childhood Disability Research, McMaster University, Hamilton, Canada; Ear Institute, University College London, London, UK; Department of Emergency Medicine, CHI St Vincent, Little Rock, Arkansas, USA; Department of Pediatrics, Life Sciences Institute, University of Michigan, Ann Arbor, MI, USA; Child Development Outpatient Clinic, Hospital da Criança Santo Antônio, Santa Casa de Porto Alegre, Porto Alegre, Brazil; Department of Psychology, Institute of Psychiatry, Psychology and Neuroscience, King’s College London, London, UK; School of Education, University of Glasgow, Glasgow, Scotland, UK; Department of Paediatrics and Child Health, University of the Witwatersrand, Johannesburg, South Africa; Department of Pediatrics and Child Health, Makerere University College of Health Sciences, Kampala, Uganda; Translational Health Research Institute, School of Medicine, Western Sydney University, Penrith, NSW, Australia; Centre for Healthy Start Initiative, Ikoyi, Lagos, Nigeria; Dr Gustavo Fricke Hospital, Neurology Infant-Juvenile, Viña del Mar, Chile; Division of Public Health, Faculty of Medicine and Health Sciences, An-Najah National University, Nablus, Palestine; Virtual Academic Unit, Children’s Directorate, Northampton General Hospital, Northampton, UK; Department of Mental Health and Substance Abuse, World Health Organization, Geneva, Switzerland; Institute of Translational Medicine, University of Liverpool, Liverpool, UK; International Centre for Evidence in Disability, London School of Hygiene & Tropical Medicine, London, UK; Eliot-Pearson Department of Child Development, Tufts University, Medford, MA, USA; Department of Population Health, London School of Economics, London, UK; The Ear Institute, University College London, London, UK; Kenya Medical Research Institute-Wellcome Trust Research Programme, Centre for Geographic Medicine Research(Coast), Kenya Medical Research Institute, KiIifi, Kenya

## Abstract

The likelihood of a newborn child dying before their fifth birthday (under-5 mortality rate) is universally acknowledged as a reflection of the social, economic, health, and environmental conditions in which children (and the rest of society) live, but little is known about the likelihood of a newborn child having a lifelong disability before their fifth birthday if he or she survives. Available data show that globally the likelihood of a child having a disability before their fifth birthday was ten times higher than the likelihood of dying (377·2 *vs* 38·2 per 1000 livebirths) in 2019. However, disability funding declined by 11·4% between 2007 and 2016, and only 2% of the estimated US$79·1 billion invested in early childhood development during this period was spent on disabilities. This funding pattern has not improved since 2016. This paper highlights the urgent need to prioritise early childhood development for the beneficiaries of global child survival initiatives who have lifelong disabilities, especially in low-income and middle-income countries, as envisioned by the Sustainable Development Goals agenda. This endeavour would entail disability-focused programming and monitoring approaches, economic analysis of interventions services, and substantial funding to redress the present inequalities among this cohort of children by 2030.

## Introduction

Children younger than 5 years have been a major focus of global health interventions since the child survival revolution started in 1982.^1^ These efforts have culminated in reducing child mortality by almost 60% since 1990.^2^ This reduction in under-5 mortality is widely acknowledged as a reflection of the social, economic, health, and environmental conditions in which children (and the rest of society) live, particularly in low-income and middle-income countries (LMICs).^3,4^ A proportion of the increased number of children surviving past their fifth birthday are children with chronic health problems and developmental disorders, which results in additional challenges for health and education systems.^5^ Children with developmental disabilities typically have permanent or lifelong functional limitations that sometimes manifest during infancy or childhood as delays in reaching age-appropriate developmental milestones.^6^ To date, the great gains in childhood survival are yet to be matched by similar improvements in the wellbeing of children with developmental disabilities, especially in countries with overburdened and fragile health-care systems.^7^

Political support for global initiatives to address this health inequality is enshrined in the Convention on the Rights of the Child,^8^ Convention on the Rights of Persons with Disabilities,^6^ subsisting Resolution of the World Health Assembly on Disability (WHA66.9),^9^ and the Sustainable Development Goals (SDGs).^10^ In particular, Sustainable Development target 4.2 requires that by 2030, “all girls and boys have access to quality early childhood development, care, and pre-primary education so that they are ready for primary education”.^10^

In this Viewpoint, we make the argument that prioritising the needs of children with developmental disabilities in global early child development initiatives is an important complement to the great efforts to reduce childhood mortality and make the case for priority consideration for children under 5 years with developmental disabilities to accelerate progress towards the 2030 end date for the SDG.

### Global risks of mortality and disability among children under 5 years

For many years, a scarcity of population-level data had constrained global initiatives and interventions for children with developmental disabilities. To bridge this gap, the Global Burden of Disease Study (GBD) periodically provides estimates of children with developmental disabilities.^11,12^ For example, in 2016, over 53 million children under 5 years globally were estimated to have developmental disabilities,^11^ with profound lifelong consequences besides the high risk of premature death, especially in LMICs where the prevalence is highest and societal attitudes towards disability are often negative and stigmatising.^13^ This estimate rises to more than 291 million for all children and adolescents younger than 20 years.^12^ Analysis of the latest GBD data^5^ shows that the prevalence of developmental disabilities is at least 75·1 per 1000 children under 5 years compared to 7·6 deaths per 1000 globally (figure). Additionally, at least 377·2 children per 1000 livebirths are likely to have a disability by the age of 5 years, compared to 38·2 who are likely to die (ie, under-5 mortality). The risk of a child being disabled is substantially higher than that of dying in all regions of the world. The gap between mortality and disability might be larger still, as our disability estimates are limited to the six conditions published in GBD 2016 (epilepsy, developmental intellectual disability, hearing loss, vision loss, autism spectrum disorder, and attention-deficit hyperactivity disorder), and do not include, for instance, the highly prevalent cerebral palsy without comorbid intellectual disability.^11^ Available evidence also suggests that children with developmental disabilities have increased risk of premature mortality or reduced life expectancy,^14^ thus underscoring the potential societal costs of failing to provide and support intervention services for the affected children and their families when it matters most. In our opinion, these data clearly suggest that any global efforts at curtailing child mortality must be complemented by appropriate support for children with developmental disabilities as they have a greater risk of poor or suboptimal physical, language, cognitive, or psychosocial development than do children without developmental disabilities.

### Globalisation of early childhood development

The need to support child development globally was first highlighted in 2000 when the US National Academy of Sciences published a landmark report on the importance of early childhood development over the entire life course.^15^ The report was based on extensive empirical evidence on the architecture of human brain development and emphasised the important role of early detection and intervention for children with developmental difficulties in the first 5 years of life and the consequent benefits to society. This was followed in 2001 by the first comprehensive report on childhood developmental disabilities in LMICs and a roadmap for action.^16^ In the same year, the International Classification of Functioning, Disability and Health was launched to standardise the evaluation of all developmental disabilities.^17^ However, the absence of explicit provisions for children with developmental disabilities in the Millennium Development Goals was a major omission that diverted requisite global attention away from the needs of these children and their families during this period.

In 2002, a special UN Resolution, for the first time, specifically called for the development and implementation of national early childhood development policies and targets for reducing disparities between children with and without developmental disabilities and special needs.^18^ However, the momentum towards child development did not begin in earnest until 2007, when the *Lancet* series on early childhood development for LMICs (and again in 2011) was launched and 200 million children under 5 years were estimated to be at risk of suboptimal development due solely to stunting or extreme poverty.^19,20^ Also, in 2007, the early childhood development knowledge network of the WHO commission on the social determinants of health highlighted the important role of the health sector as the intersection of effective global initiatives for early childhood development.^21^ However, children considered to be at risk of suboptimal development were defined on the basis of stunting and poverty, and children with developmental disabilities were excluded because of the scarcity of population-level disability data from LMICs at a time when no data implied no problem, and therefore no action.^19^ This data gap was partly bridged in 2013, by UNICEF’s annual report on the state of the world’s children devoted exclusively to children with disabilities.^13^ Sustained advocacy by the disability community ensured that the emerging SDGs in 2015 were more disability inclusive than the preceding Millennium Development Goals. Seven targets explicitly refer to people with disabilities, eight to people in vulnerable situations, and one focuses on non-discrimination.^10^ The pledge to “leave no one behind”^10^ also implied that the needs of children with developmental disabilities would no longer be overlooked.

In 2017, a third *Lancet* series on early childhood development updated the estimate of children at risk of suboptimal development as 250 million, still based on stunting and poverty.^22^ This data formed the foundation of the Nurturing Care Framework for early childhood development jointly promoted by UNICEF, WHO, and the World Bank Group since 2018.^2,3^ The Nurturing Care Framework is focused on a wide range of crosscutting interventions for all children from conception to age 3 years, including health promotion, nutrition, responsive caregiving, early learning, security, and safety. It is now actively promoted as the de-facto early childhood development programme for LMICs. However, although the Nurturing Care Framework acknowledges that children with developmental disabilities have greater needs than their peers without developmental disabilities, it does not emphasise priority attention for these children.^23^ Additionally, whereas the SDG’s agenda pledges to leave no one behind, in the UN flagship report on disability no progress was reported on SDG 4.2 regarding early childhood development for children under 5 years with developmental disabilities.^24^

### Prioritising support for children under 5 years with developmental disabilities

The remarkable achievement in reducing child mortality globally since 1990 was made possible by the concerted efforts of governments and several non-state actors in global health that started with identifying the major contributors to newborn and child mortality globally and the requisite interventions. As a result, infectious diseases (including pneumonia, diarrhoea, and malaria), along with prematurity, birth asphyxia and trauma, and congenital anomalies were prioritised. This attracted substantial global funding for strengthening health-care systems to deliver the requisite services. For example, the Development Assistance for Health (the financial and in-kind contributions) transferred through major international development agencies to LMICs for new-born and child survival rose from $2·0 billion in 2000, to $7·6 billion in 2016, totalling $76·3 billion.^25^ In 2019, $8·5 billion was spent on child survival and development representing about 21% of total Development Assistance for Health.

We acknowledge that global initiatives for improved maternal and child health services and the strengthening of national health systems might be relevant in addressing childhood developmental disabilities. However, the massive global investments in reducing child mortality in LMICs has yet to reflect the disturbing disparity between under-5 mortality and under-5 disability. For example, an analysis of the available data on global funding for early childhood development showed that total development assistance increased by 121% between 2007 and 2016, an average annual increase of 8·3%.^26^ However, a disaggregation by the Nurturing Care Framework activity domains showed improvement in disbursements for nutrition and growth (24·5%), responsive care (22·0%), early learning (21·9%), security and safety (7·9%), and good health (6·5%), but a decline for disability (–11·4%). In fact, of the estimated $79·1 billion disbursed as development assistance for early childhood development, $61·9 billion (78% of the total) was for health and nutrition and $0·7 billion (the least amount; 2% of the total) was for disability. There is no evidence to suggest that the global funding pattern for child health has been more disability-inclusive since 2016. Although the core package of services offered by the Nurturing Care Framework are essential for all children, they are vastly inadequate to address the most pressing needs of children with developmental disabilities.^23^ More crucially, because these children have the greatest risk of suboptimal outcomes, especially because of access barriers to early learning opportunities and higher risks of being exposed to abuse and neglect, stigma, and discrimination,^13^ they should be prioritised in any global early childhood development initiative especially where resources and capacity to serve all children are scarce.

Early detection and timely intervention are the cornerstone of any effective early childhood development programme and more so for children with developmental disabilities.^27^ The Convention on the Rights of Persons with Disabilities, for example, specifically requests the development of comprehensive rehabilitation and support services by relevant authorities that promote maximum independence and full participation in all aspects of life and urges such services to “begin at the earliest possible stage”.^6^ The academic literature offers a wide range of possible screening tools that can assist caregivers and parents to navigate the diagnostic maze of uncovering the particular needs of a child with disability.^28^ There are potentially cost-effective rehabilitation strategies that can improve developmental outcomes in children with developmental disabilities if adequately funded.^7,29,30^ The required services cut across several medical and non-medical disciplines with the health sector frequently being the primary gateway for most children and their families. A growing number of stakeholders and global health actors, including international and local non-government organisations, have also developed community-based networks to deliver services. However, a recent assessment from WHO and UNICEF shows that the unmet needs of children and adolescents with disability are substantial and increasing steadily because services do not exist in most LMICs or have not been expanded despite many more children requiring care.^7^ For example, whereas newborn screening programmes to facilitate early detection of children with developmental disabilities are routinely offered in all high-income countries, it is not the case in LMICs. Services that exist are often fragmented, under resourced, of poor quality, not affordable, and confined to urban areas. Meanwhile, besides the elevated risk of premature death over the life course,^14^ evidence from LMICs suggest that care providers and mothers consider mild disability as somewhat better than death, moderate disability as bad as death, and severe and profound disability as far worse than death.^31^ This situation is likely to worsen if services for children with developmental disabilities are not prioritised globally.

### Facilitating sustainable action plans for children under 5 years with developmental disabilities

Many LMICs, and donor agencies rely on WHO and UNICEF for guidance on the priorities and best practices for child health and development. Although several reports on childhood developmental disabilities have been published by WHO and UNICEF, an authoritative roadmap for supporting children with developmental disabilities is not yet available. Economic analysis of screening and intervention services for specific developmental disabilities that can be widely implemented in LMICs has not yet been reported. It is, therefore, important to accelerate the ongoing efforts by WHO, UNICEF, and their partners to produce a global report on developmental disabilities that truly reflects the extensive body of knowledge from LMICs. Countries should be supported to do pilot programmes to determine context-specific adaptations of the emerging generic guidelines and recommendations. Health-care systems at the primary, secondary, and tertiary levels need to be restructured and strengthened to facilitate the delivery of rehabilitation services. Development of low-cost technologies and other evidence-based approaches to support family-centred services for children with developmental disabilities is also necessary. Sociocultural barriers to the uptake of services must be addressed through transparent community and stakeholder engagements, including disabled people’s organisations and experts from LMICs. Examples of successful early childhood development programmes in LMICs that have included services for children with developmental disabilities (eg, the Rashtriya Bal Swasthya Karyakram initiative across India aimed at early identification and intervention for children with developmental disabilities from birth to age 18 years)^32^ need to be identified and replicated widely.

The COVID-19 pandemic has presented unprecedented challenges for children with developmental disabilities and their families.^33,34^ The effect in shifting the traditional pathway to service delivery towards virtual interactions, including online coaching for families, must be recognised.^33–35^ Although restrictions might be phased out in countries with low rates of infection and improved vaccination coverage, it is unlikely that the traditional management practices for childhood developmental disabilities will be fully restored, thus necessitating special investment considerations for appropriate technologies for outpatient screening, diagnostic, and ongoing family-centred support.

### Tracking global progress until 2030

SDG 4.2 explicitly mandates tracking progress towards early childhood development by monitoring “the proportion of children under 5 years who are developmentally on track in health, learning and psychosocial wellbeing”.^10^ Conceptually, children who “are developmentally on track”^10^ are those without developmental disabilities or developmental delays, based on standardised milestones of development in early childhood. However, children with developmental disabilities are unlikely to be developmentally on track in all functional domains over the life course by any quantitative measure. More crucially, children with developmental disabilities now compete unfairly for attention with those perceived to be at risk of developmental disabilities because of a self-limiting developmental or constitutional delay in reaching age-specific milestones. The current practice of aggregating children with a broad range of transient or reversible developmental delays not usually associated with a chronic condition or a specific diagnosis, with children with developmental disabilities needs to be revisited based on differences in the referral pathways to care and the duration of the required support. This consideration should not overlook the fact that some developmental disabilities might present initially as developmental delays before diagnosis is confirmed. For example, the US Individuals with Disabilities Education Act mandates a clear and rigorous definition of the term “developmental delay”^10^ that will be used to appropriately identify infants and toddlers in need of services under the Act.^36^ Additionally, amorphous concepts such as “loss of developmental potential”, “risk of not realising developmental potential”,^19,20,22,23^ and other proxy measures of child growth and development have by design or default diverted attention from children with developmental disabilities. These proxy measures, including stunting and poverty, are usually distal to many developmental outcomes and minimally responsive to programmatic interventions.^37^ Furthermore, most intervention studies on early childhood development especially in LMICs exclude children with developmental disabilities as additional considerations might be required, and children with developmental disabilities might be viewed as complicating the assessment of intervention effectiveness when using developmental progress as the primary outcome.^30,38^

Investing in early childhood development for children with developmental disabilities is likely to improve their functioning, quality of life, and capabilities, as well as the quality of life and mental health of their carers. The basic principles of neuroscience and the econometrics of human capital development also show that early and effective intervention for the most vulnerable children will generate the greatest financial payback.^39^ In addition, the financial implications of the failure to take appropriate and timely action are considerable because childhood disability imposes a substantial economic effect on families, health-care systems, and societies.^40^ The annual costs of childhood disability worldwide are estimated at US$450–69 500. The lifetime costs per child ranges from approximately $41 000 to $4 300 000, worldwide. Thus, an appropriately focused global early childhood development policy and programming are urgently needed to accelerate progress in addressing the priority needs of children with developmental disabilities. More importantly, the proportion of children receiving early detection and intervention services in each country should be monitored as a crucial performance indicator. This metric can assist in determining actionable global targets for early intervention enrolment to complement existing SDG targets for under-5 mortality and undernutrition.^10^ Based on the evidence presented in this Viewpoint, it is politically, economically, and morally imperative that a substantial proportion of global health funding for newborn and child health, as a priority, be channelled to disability focused early childhood development programmes.

## Conclusion

Globally, the risk of a child having a disability is at least ten times that of dying before the fifth birthday. Disability also increases the risk of premature death and reduced life expectancy. However, there has been little progress towards prioritising the needs of children with developmental disabilities in global early childhood development initiatives. Targeted improvements in child mortality and disability are not mutually exclusive moral imperatives, especially in LMICs. It is, therefore, necessary that children with developmental disabilities are rapidly repositioned from the margins to the centre of global development strategies for child health and wellbeing in line with the subsisting and unfulfilled commitments of UN member states since 2002. This would require, among other initiatives, that global funding for child health and early childhood development henceforth includes specific and significant provisions for children with developmental disabilities. We hold the view, with other stakeholders in the global health community, that it is the right thing to do.

## Figures and Tables

**Figure:**
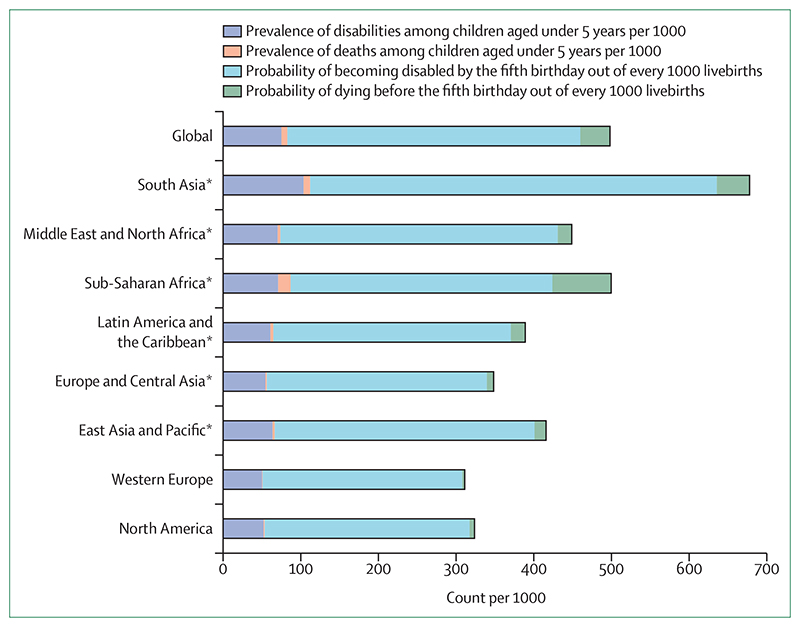
Global and regional estimates of disability and mortality among children aged under 5 years in 2019 Data source is the Global Health Data Exchange. *Countries were grouped by World Bank region.

## Key messages

- Globally, the likelihood of a child having a disability is at least ten times higher than that of dying before the fifth birthday
- Children with developmental disabilities have the greatest risk of poor development in the early crucial years preparatory to inclusive primary school enrolment
- Whereas substantial global funding has been committed to the reduction in child mortality since 2000, investment in child disability declined by 11·4% between 2007 and 2016
- Targeted reductions in under-5 mortality especially in low-income and middle-income countries should be accompanied with complementary investment in addressing the needs of the beneficiaries of child survival programmes with lifelong disabilities
- Global initiatives for early childhood development should be prioritised for children with developmental disabilities from birth as envisioned by the Sustainable Development Goals and supported by robust scientific evidence for optimal early intervention
